# Influence of high‐intensity interval training to exhaustion on the directional sensitivity of the cerebral pressure‐flow relationship in young endurance‐trained men

**DOI:** 10.14814/phy2.15384

**Published:** 2022-07-13

**Authors:** Faezeh Abbariki, Marc‐Antoine Roy, Lawrence Labrecque, Audrey Drapeau, Sarah Imhoff, Jonathan D. Smirl, Patrice Brassard

**Affiliations:** ^1^ Department of Kinesiology, Faculty of Medicine Université Laval Québec City Québec Canada; ^2^ Research Center of the Institut Universitaire de Cardiologie et de Pneumologie de Québec Québec City Québec Canada; ^3^ Cerebrovascular Concussion Laboratory, Faculty of Kinesiology University of Calgary Calgary Alberta Canada; ^4^ Sport Injury Prevention Research Centre, Faculty of Kinesiology University of Calgary Calgary Alberta Canada; ^5^ Human Performance Laboratory, Faculty of Kinesiology University of Calgary Calgary Alberta Canada; ^6^ Hotchkiss Brain Institute University of Calgary Calgary Alberta Canada; ^7^ Integrated Concussion Research Program University of Calgary Calgary Alberta Canada; ^8^ Alberta Children's Hospital Research Institute University of Calgary Calgary Alberta Canada; ^9^ Libin Cardiovascular Institute of Alberta University of Calgary Alberta Canada; ^10^ Concussion Research Laboratory, Faculty of Health and Exercise Science University of British Columbia Kelowna British Columbia Canada

**Keywords:** high‐intensity interval training, hysteresis, mean arterial pressure, repeated squat‐stands

## Abstract

We previously reported subtle dynamic cerebral autoregulation (dCA) alterations following 6 weeks of high‐intensity interval training (HIIT) to exhaustion using transfer function analysis (TFA) on forced mean arterial pressure (MAP) oscillations in young endurance‐trained men. However, accumulating evidence suggests the cerebrovasculature better buffers cerebral blood flow changes when MAP acutely increases compared to when MAP acutely decreases. Whether HIIT affects the directional sensitivity of the cerebral pressure‐flow relationship in these athletes is unknown. In 18 endurance‐trained men (age: 27 ± 6 years, VO_2_max: 55.5 ± 4.7 ml·kg^−1^·min^−1^), we evaluated the impact of 6 weeks of HIIT to exhaustion on dCA directionality using induced MAP oscillations during 5‐min 0.05 and 0.10 Hz repeated squat‐stands. We calculated time‐adjusted changes in middle cerebral artery mean blood velocity (MCAv) per change in MAP (ΔMCAv_T_/ΔMAP_T_) for each squat transition. Then, we compared averaged ΔMCAv_T_/ΔMAP_T_ during MAP increases and decreases. Before HIIT, ΔMCAv_T_/ΔMAP_T_ was comparable between MAP increases and decreases during 0.05 Hz repeated squat‐stands (*p* = 0.518). During 0.10 Hz repeated squat‐stands, ΔMCAv_T_/ΔMAP_T_ was lower during MAP increases versus decreases (0.87 ± 0.17 vs. 0.99 ± 0.23 cm·s^−1^·mmHg^−1^, *p* = 0.030). Following HIIT, ΔMCAv_T_/ΔMAP_T_ was superior during MAP increases over decreases during 0.05 Hz repeated squat‐stands (0.97 ± 0.38 vs. 0.77 ± 0.35 cm·s^−1^·mmHg^−1^, *p* = 0.002). During 0.10 Hz repeated squat‐stands, dCA directional sensitivity disappeared (*p* = 0.359). These results suggest the potential for HIIT to influence the directional sensitivity of the cerebral pressure‐flow relationship in young endurance‐trained men.

## Graphical abstract

The main findings of the current study indicate there is a frequency‐dependent effect of 6 weeks of high‐intensity interval training to exhaustion on the cerebral pressure‐flow relationship. Specifically, we report the disappearance of directional sensitivity at 0.10 Hz repeated squat‐stands and the appearance of an inverted directionality pattern at 0.05 Hz repeated squat‐stands following the training intervention in young endurance‐trained men.

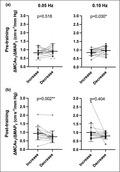

## 1 INTRODUCTION

Dynamic cerebral autoregulation (dCA) describes the ability of the cerebral vessels to react to rapid blood pressure changes (Aaslid et al., 1989). Although regular aerobic exercise is associated with multiple cerebrovascular adaptations (Bliss et al., 2021; Lucas et al., 2015; Smith et al., 2021), the direct impact of exercise training on the cerebral pressure‐flow relationship is currently largely unexplored.

Improvements in cardiorespiratory fitness associated with aerobic exercise training are thought to have robust protective effects on the cerebrovascular function (Ainslie et al., 2008; Bailey et al., 2013; Smith et al., 2021). Higher cardiorespiratory fitness is often related to higher resting middle cerebral artery mean blood velocity (MCAv) (Ainslie et al., 2008), cerebrovascular reactivity to carbon dioxide (CO_2_), and lower cerebrovascular resistance index (Smith et al., 2021). However, cardiorespiratory fitness may have less of an impact on dCA (Maxwell et al., 2022), and whether aerobic exercise training leads to improvements in dCA remains equivocal. Indeed, improving cardiorespiratory fitness with an exercise training program may enhance dCA, but only with the added stimulus of a cold environment, as thermoneutrality appears to negate it (Miller et al., 2022). Furthermore, studies suggest dCA is either maintained (Aengevaeren et al., 2013; Ichikawa et al., 2013) or less efficient in endurance‐trained athletes than in sedentary controls (Labrecque et al., 2017; Lind‐Holst et al., 2011). Additionally, the different ways of assessing dCA used across these studies warrant caution as metrics may not correlate with each other (Tzeng et al., 2012). While the aforementioned literature included individuals with a history of mostly moderate‐intensity endurance training, results focusing on both a controlled exercise intervention and a higher training intensity are relatively scarce.

High‐intensity interval training (HIIT) is generally referred to as exercise bouts performed at intensities of 85% to 95% of maximal heart rate and interspersed with rest or active light exercise recovery periods (Calverley et al., 2020; MacInnis & Gibala, 2017). Extensive research has been conducted on HIIT, indicating its relevance in clinical and physiological research with improvements in cardiorespiratory fitness beyond moderate‐intensity continuous training (Calverley et al., 2020; MacInnis & Gibala, 2017; Weston et al., 2014). Although HIIT presents an interesting stimulus for the cardiovascular system, a limited number of studies have focused on its effects on the cerebrovascular system (reviewed in Whitaker et al., 2020). Indeed, recent work by Whitaker et al. indicated more research is needed to confirm the acute (Whitaker et al., 2022) and chronic (Whitaker et al., 2020) effects of HIIT on the cerebrovascular function.

We have previously reported subtle alterations in dCA following 6 weeks of HIIT to exhaustion (3 sessions per week at 85% or 115% of maximal aerobic power) using transfer function analysis (TFA) on forced mean arterial pressure (MAP) oscillations in young endurance‐trained men (Drapeau et al., 2019). Specifically, TFA phase at 0.10 Hz repeated squat‐stands was decreased after HIIT, irrespective of training intensity. TFA represents an analysis which does not take the direction of MAP changes into consideration. Using time‐domain analyses, it has been suggested that dynamic cerebral pressure‐flow responses should be analyzed in each MAP direction separately (Labrecque, Smirl, et al., 2022). Accumulating evidence suggests the cerebrovasculature is better adapted to buffer cerebral blood flow (CBF) changes when MAP transiently increases compared to when MAP transiently decreases (Brassard et al., 2017; Labrecque, Burma, et al., 2022; Labrecque et al., 2021; Panerai et al., 2018; Roy et al., 2022; Tzeng et al., 2010). Doing so, one study suggests endurance‐trained athletes show a similar pattern of dCA directional sensitivity as sedentary controls (Roy et al., 2022). However, unlike other endurance training programs, HIIT‐related repetitive high MAP and subsequent cerebral blood velocity fluctuations (Labrecque et al., 2020) may pose their own risk to cerebrovascular integrity as studies raised the concern of a potential breakthrough of the blood–brain barrier (Bailey et al., 2011; Lucas et al., 2015). Therefore, knowing whether MAP surges remain better buffered than transient MAP reductions as a result of repeated HIIT sessions is worth investigating.

The present analysis is part of a larger data set on which dCA has been previously analyzed using TFA, thus without taking MAP direction into consideration. Accordingly, the aim of the current study was to expand on the matter by assessing the effect of HIIT to exhaustion on the directional sensitivity of the cerebral pressure‐flow relationship in these young endurance‐trained men. We hypothesized 6 weeks of HIIT to exhaustion would maintain the directional sensitivity of the cerebral pressure‐flow relationship in endurance‐trained young men.

## 2 MATERIALS AND METHODS

### 2.1 Ethics and informed consent

The current study was approved by the *Comité d'éthique de la recherche de l'Institut universitaire de cardiologie et de pneumologie de Québec‐Université Laval* (CER: 20869) in accordance to the principles established in the Declaration of Helsinki (except for registration in a database). Prior to the investigation, all participants provided informed written consent.

### 2.2 Participants

Nineteen endurance‐trained men were recruited to conduct this study. All participants had a history of exercise of 5–12 h per week, for at least 2 years. Also, they had no history of cerebrovascular or cardiovascular disease. Athletes with different backgrounds in endurance sports participated in this study; participants were cross‐country skier (*n* = 1), mountain bikers (*n* = 2), triathletes (*n* = 7), and road cyclists (*n* = 9) (Paquette et al., 2017).

### 2.3 Experimental protocol

This analysis is part of a larger study conducted by Paquette et al. (2017), which examined the influence of submaximal and supramaximal training on determinants of endurance performance (Paquette et al., 2017). An important feature of our training protocol was that all participants in each session performed HIIT until exhaustion. Indeed, participants who performed submaximal (85% of maximal aerobic power) and supramaximal HIIT (115% of maximal aerobic power) were matched for total effort (e.g., they were asked to perform each exercise repetition to exhaustion) rather than for total work, which is closer to what athletes typically do when performing high‐intensity interval sessions at different intensities (Seiler et al., 2013). Using the same cohort of participants, Drapeau et al. (2019) examined the effect of 6 weeks of HIIT (at both 85% and 115% of maximal aerobic power) on dCA evaluated with TFA (Drapeau et al., 2019). The current analysis represents a secondary analysis, based on the same larger data set used to examine dCA directional sensitivity. All participants were asked to abstain from consuming caffeine and alcoholic beverages for a period of 24 h, and to avoid exercise training for at least 12 h (Burma et al., 2020). Data collection of all participants was done in the same order and the the visit sessions (before and following the training intervention) were at least 48 h apart. The post‐training testing sessions were repeated 48–96 h following the end of the 6‐week training program.

### 2.4 Training interventions

Training sessions were completed three times a week for 6 weeks with an interval of 48–72 h between each session. Participants were allowed to exercise on their own at a volume similar to the pre‐study exercise volume of low or moderate intensity (monitored with a weekly physical activity logbook). However, HIIT was prohibited.

Participants were randomly divided into HIIT 85% (*n* = 9) and HIIT 115% (*n* = 10) groups. The first training group performed repeated effort bouts of 1–7 min at 85% maximal aerobic power, with an active recovery duration corresponding to half the effort bout at 50% of maximum aerobic power or 150 W if the maximum power was less than 300 W. The second training group performed repeated effort bouts of 30 s to 1 min at 115% maximum aerobic power, with an active recovery duration corresponding to twice the effort bout at 50% of maximum aerobic power or 150 W if the maximum power is less than 300 W. Since the focus of this original study was on the intensity of exercise (Paquette et al., 2017), the duration of intervention sessions was varied over a period of 6 weeks in order to reduce monotony. Refer to Paquette et al. (2017) for more details on training sessions and interventions (Paquette et al., 2017).

We have previously showed this HIIT program improves maximal oxygen uptake (VO_2_max) and anaerobic power (Paquette et al., 2017) and influences dCA quantified using TFA (Drapeau et al., 2019). Importantly, these adaptations following the 6‐week HIIT program was independent of training intensity. Accordingly, data from the two groups were pooled for the current analysis.

### 2.5 Visit 1

Height and body mass of all participants were measured (Table 1). Then, resting hemodynamics of all participants was recorded for 10 min in the supine position. Heart rate was measured by 5‐lead ECG and blood pressure was measured using arterial volume clamping (Nexfin, Edwards Lifesciences, Ontario, Canada) on the middle finger of the right hand. MCAv was monitored with a 2 MHz pulsed transcranial Doppler ultrasound (Doppler Box, Compumedics DWL USA, Inc.). End‐tidal partial pressure of carbon dioxide (P_ET_CO_2_) was recorded by a gas analyzer (Breezesuite, MedGraphics Corp.) during baseline rest and repeated squat‐stand maneuvers. The gas analyzer was calibrated according to the manufacturer's instructions before each measurement.

**TABLE 1:** Baseline characteristics and resting values before and after training

Variables	*N*	Before training	After training	*p* value	Cohen's *d* (95% CI)
Age (years)	18	27 ± 5			
Height (m)	18	1.76 ± 0.1			
Body mass (kg)	17	71.5 ± 10.4	70.4 ± 10.3	0.057	0.50 (0.31–1.12)
Maximal oxygen consumption (ml·kg^−1^·min^−1^)	17	55.5 ± 4.7	58.7 ± 4.1	0.008	0.74 (0.21–1.80)
Heart rate (bpm)	18	54 ± 8	51 ± 7	0.002	0.87 (0.60–1.65)
MAP (mmHg)	18	78 ± 9	81 ± 12	0.377	0.21 (−0.63–0.37)
MCAv (cm·s^−1^)	18	66 ± 9	67 ± 11	0.196	0.21 (−0.97–0.23)
P_ET_CO_2_ (mmHg)	12	44 ± 4	46 ± 3	0.069	0.58 (0.15–1.18)

*Note*: Variables were analyzed within individual before and after 6 weeks of high‐intensity interval training using paired *t*‐tests. *p* < 0.05 is considered statistically significant. Cohen's *d* with 95% confidence intervals (CI) evaluates effect sizes: Negligible below 0.20, small from 0.20 to 0.50, moderate from 0.50 to 0.80 and large above 0.80.

### 2.6 Repeated squat‐stand maneuvers

Familiarization of participants with the repeated squat‐stand maneuvers was done through mirroring the movement of the experimenter before the main maneuver. Participants performed repeated squat‐stand maneuvers in two steps in a randomized order, each step lasting 5 min, at 0.05 Hz (10 s squat position and 10 s standing position) and 0.10 Hz (5 s squat position and 5 s standing position) separated by 5 min of rest (Brassard et al., 2017).

### 2.7 Visit 2

VO_2_max was measured on an electromagnetically braked upright cycle ergometer (Corival, Lode, Netherlands) using a progressive ramp protocol. In brief, the maximal exercise protocol was as followed: After a 3‐min rest, all participants performed a 1‐min warm‐up of unloaded cycling, then executed an incremental ramp protocol (25 or 30 W·min^−1^) to exhaustion. VO_2_max was defined with the highest 30 s mean VO_2_, concomitant with a respiratory exchange ratio of ≥1.15 (Paquette et al., 2017).

### 2.8 Visit 3

As previously described, maximal aerobic power was assessed using an intermittent 5‐min stage cycling test on the same cycle ergometer in order to set intensity of the training sessions (Paquette et al., 2017).

### 2.9 Data acquisition

An analog‐to‐digital converter (Powerlab 16/30 Ml880; ADInstruments) converted and stored at 1 kHz all signals. Subsequent analysis was performed with a free version of a commercial software (LabChart version 8.1.8; ADInstruments). P_ET_CO_2_ was acquired separately with Breeze Suite (MedGraphics Corp.), and time‐aligned with the other signals.

### 2.10 Data analysis

To characterize the impact of 6 weeks of HIIT on the directional sensitivity of the cerebral pressure‐flow relationship, we utilized a time‐adjusted ratio as described by our team for each squat‐stand transition during the 5 min of repeated squat‐stands at both frequencies. For a more in‐depth explanation of the current data analysis, we refer readers to this previous work (Labrecque et al., 2021).

The time‐adjusted absolute changes (ΔMCAv_T_/ΔMAP_T_) during transient decreases in MAP (from maximum to minimum) were calculated as follows (Labrecque et al., 2021):

$$\frac{\frac{\left({\text{MCAv}}_{\min}-{\text{MCAv}}_{\max}\right)}{\left({\text{Time}}_{\text{MCAvmin}}-{\text{Time}}_{\text{MCAvmax}}\right)}}{\frac{\left({\mathrm{MAP}}_{\min}-{\mathrm{MAP}}_{\max}\right)}{\left({\text{Time}}_{\text{MAPmin}}-{\text{Time}}_{\text{MAPmax}}\right)}}=\frac{\frac{∆\text{MCAv}}{∆{\text{Time}}_{\text{MCAv}}}}{\frac{∆\mathrm{MAP}}{∆{\text{Time}}_{\mathrm{MAP}}}}=\frac{∆{\text{MCAv}}_{T}}{∆{\mathrm{MAP}}_{T}}.$$

ΔMCAv_T_/ΔMAP_T_ changes during transient increases in MAP (from minimum to maximum) were calculated as follows (Labrecque et al., 2021):

$$\frac{\frac{\left({\text{MCAv}}_{\max}-{\text{MCAv}}_{\min}\right)}{\left({\text{Time}}_{\text{MCAvmax}}-{\text{Time}}_{\text{MCAvmin}}\right)}}{\frac{\left({\mathrm{MAP}}_{\max}-{\mathrm{MAP}}_{\min}\right)}{\left({\text{Time}}_{\text{MAPmax}}-{\text{Time}}_{\text{MAPmin}}\right)}}=\frac{\frac{∆\text{MCAv}}{∆{\text{Time}}_{\text{MCAv}}}}{\frac{∆\mathrm{MAP}}{∆{\text{Time}}_{\mathrm{MAP}}}}=\frac{∆{\text{MCAv}}_{T}}{∆{\mathrm{MAP}}_{T}}.$$

To evaluate whether repeated squat‐stands induced changes in P_ET_CO_2_, an averaged P_ET_CO_2_ of the first and last five breaths for each maneuver (0.05 and 0.10 Hz) were calculated (Drapeau et al., 2019; Labrecque, Burma, et al., 2022; Labrecque et al., 2019, 2021; Perry et al., 2019; Roy et al., 2022).

### 2.11 Statistical analysis

The Shapiro–Wilk test was used to confirm normal distribution of data. Paired *t*‐tests were used to compare resting values, as well as changes in P_ET_CO_2_ during repeated squat‐stands, before and after training. The presence of a directional sensitivity of the cerebral pressure‐flow relationship was assessed at each repeated squat‐stand frequency, before and after HIIT, by using paired *t*‐tests to compare increases and decreases in MAP within individuals. Changes observed during repeated squat‐stands to calculate directionality, herein hemodynamic changes (∆MCAv, ∆MAP) and time intervals (∆Time_MCAv_, ∆Time_MAP_), were analyzed at each frequency with a two‐way (MAP direction and training) mixed‐model ANOVA. Multiple comparisons used Bonferonni correction. Effect sizes were reported using the generalized eta‐squared ($\eta_{G}^{2}$) for all ANOVA analyses (≤0.02 negligible, [0.03, 0.13] small, [0.13, 0.25] moderate, ≥0.26 large) (Bakeman, 2005) and Cohen's *d* for comparisons between means (≤0.19 negligible, [0.20, 0.49] small, [0.50, 0.79] moderate, ≥0.80 large) (Lakens, 2013). Data are expressed as means ± SD. *p* value <0.05 were considered statistically significant for all tests. Statistical analyses were performed using Graphpad prism (version 9.3.1) and effect sizes were calculated using Rstudio (version 4.1.1).

## 3 RESULTS

Of the 19 participants initially recruited, data are presented for 18 participants. One of the participants was excluded since post‐training tests were not completed. Of those 18 participants, one participant's pre‐training data were excluded at 0.05 Hz repeated squat‐stands because blood pressure and MCAv tracings did not show the identifiable oscillations needed for our analyses. Post‐training data from three participants were excluded at 0.10 Hz repeated squat‐stands because of technical problems in blood pressure acquisition. P_ET_CO_2_ data from 6 participants were not included owing to technical reasons. Table 1 shows participants' pre‐ and post‐training characteristics and resting values in the supine position.

### 3.1 Baseline characteristics and resting values

The 6‐week HIIT protocol did not modify body mass (*p* = 0.057, *d* = 0.50 [CI: 0.31–1.12] [small]) and improved VO_2_max by 5.8% (*p* = 0.008, *d* = 0.74 [CI: 0.21–1.80] [moderate]). The training program significantly decreased resting HR by 5.6% (*p* = 0.002, *d* = 0.87 [CI: 0.60–1.65] [large]), while other hemodynamic values did not change after the HIIT program (Table 1). These results were previously reported, although with participants' HIIT intensity as an additional factor in the analysis (Drapeau et al., 2019; Paquette et al., 2017).

### 3.2 Hemodynamic changes and time intervals during squat‐stands

Data regarding hemodynamic changes during repeated squat‐stands are shown in Table 2. Driven absolute changes in MAP and MCAv averaged across 5 min of repeated squats‐stands did not differ between MAP increases and decreases. MCAv changes were of significantly lower amplitude during 0.05 Hz (training: *F*_(1, 16)_ = 6.58, *p* = 0.010, $\eta_{G}^{2}$ = 0.05 [small]) and 0.10 Hz (training: *F*_(1, 15)_ = 9.69, *p* = 0.007, $\eta_{G}^{2}$ = 0.09 [small]) repeated squat‐stands after the training protocol. At 0.05 Hz repeated squat‐stands, those changes in MCAv were of shorter duration when MAP increased, irrespective of whether participants had gone through the HIIT protocol or not (MAP direction: *F*_(1, 16)_ = 14.09, *p* < 0.001, $\eta_{G}^{2}$ = 0.35 [large]). There was an interaction effect of training and MAP direction at 0.05 Hz repeated squat‐stands for time intervals of MAP (*F*_(1, 16)_ = 8.23, *p* < 0.001, $\eta_{G}^{2}$ = 0.17 [moderate]). Post‐hoc analyses revealed quicker changes of MAP increases before HIIT (*p* < 0.001, *d* = 1.61 [CI: 1.15–2.61] [large]), but not after HIIT (*p* = 0.912, *d* = 0.21 [CI: −0.79 to 0.26] [small]). Additionally, increases in MAP became significantly longer after training (*p* = 0.032, *d* = 0.71 [CI: 0.32–1.19] [moderate]), while decreases in MAP did not become shorter (*p* = 0.069), despite a moderate effect size (*d* = 0.67 [CI: 0.27–1.23]). Significant interaction effects were observed at 0.10 Hz repeated squat‐stands for MAP changes (*F*_(1, 15)_ = 3.55, *p* = 0.023, $\eta_{G}^{2}$ < 0.02 [negligible]) and time intervals of MCAv (*F*_(1, 15)_ = 3.46, *p* = 0.018, $\eta_{G}^{2}$ = 0.09 [small]), however no post hoc differences were isolated.

**TABLE 2:** Averaged hemodynamic changes and time intervals during squat‐stands before and after training

Training	Pre‐training		Post‐training		*p*‐value		
MAP direction	Increase	Decrease	Increase	Decrease	Training	Direction	Interaction
0.05 Hz	*n* = 17		*n* = 18				
∆MCAv (cm·s^−1^)	32 ± 14	32 ± 14	26 ± 12	25 ± 12	0.010	0.419	0.623
∆MAP (mmHg)	37 ± 15	38 ± 14	34 ± 13	34 ± 13	0.172	0.988	0.076
∆TimeMCAv (s)	9.1 ± 1.3	10.8 ± 1.2	8.8 ± 1.5	11.2 ± 1.5	0.959	<0.001	0.266
∆TimeMAP (s)	8.7 ± 0.8	11.1 ± 0.7^a^	9.7 ± 1.3^b^	10.2 ± 1.1^c^	0.789	<0.001	<0.001
0.10 Hz	*n* = 18		*n* = 16				
∆MCAv (cm·s^−1^)	29 ± 11	29 ± 11	24 ± 9	24 ± 9	0.007	0.120	0.704
∆MAP (mmHg)	34 ± 14	34 ± 14	30 ± 12	30 ± 12	0.090	0.786	0.023
∆TimeMCAv (s)	5.2 ± 0.5	4.8 ± 0.5	4.8 ± 0.9	5.2 ± 0.8	0.953	0.802	0.018
∆TimeMAP (s)	4.9 ± 0.5	5.1 ± 0.5	5.1 ± 0.6	4.9 ± 0.6	0.918	0.716	0.070

*Note*: Directional component values are presented as 5‐min averages of squat–stands data at each frequency for acute mean arterial pressure (MAP) increases and MAP decreases in all training groups. Values are means ± SD. *p*‐values were determined with a mixed two‐way model ANOVA. Bonferonni correction on multiple comparisons with Cohen's *d* effect size [95% confidence intervals].

^a^ Pre‐training increase versus decrease, *p* < 0.001, *d* = 1.61 [CI: 1.15–2.61] (large).

^b^ Increase pre‐ versus post‐training, *p* = 0.032, *d* = 0.71 [CI: 0.32–1.19] (moderate).

^c^ Decrease pre‐ versus post‐training, *p* = 0.069, *d* = 0.67 [CI: 0.27–1.15] (moderate).

P_ET_CO_2_ changes were assessed in 0.05 Hz (*n* = 6) and 0.10 Hz (*n* = 12) repeated squat‐stands. By calculating the difference between the first 5 breaths and the last 5 breaths of the 5‐min repeated squat‐stands, changes in P_ET_CO_2_ did not significantly differ between pre‐ and post‐training (0.05 Hz: +2.7 ± 1.7 vs. −0.2 ± 3.4, *p* = 0.093, *d* = 0.85 [CI: 0.28–3.80] [large]; 0.10 Hz: +2.7 ± 1.7 vs. +2.2 ± 3.0, *p* = 0.640, *d* = 0.20 [CI: −0.71 to 2.01] [negligible]) (Drapeau et al., 2019).

### 3.3 Directional sensitivity of the cerebral pressure‐flow relationship before exercise training

**FIGURE 1:**
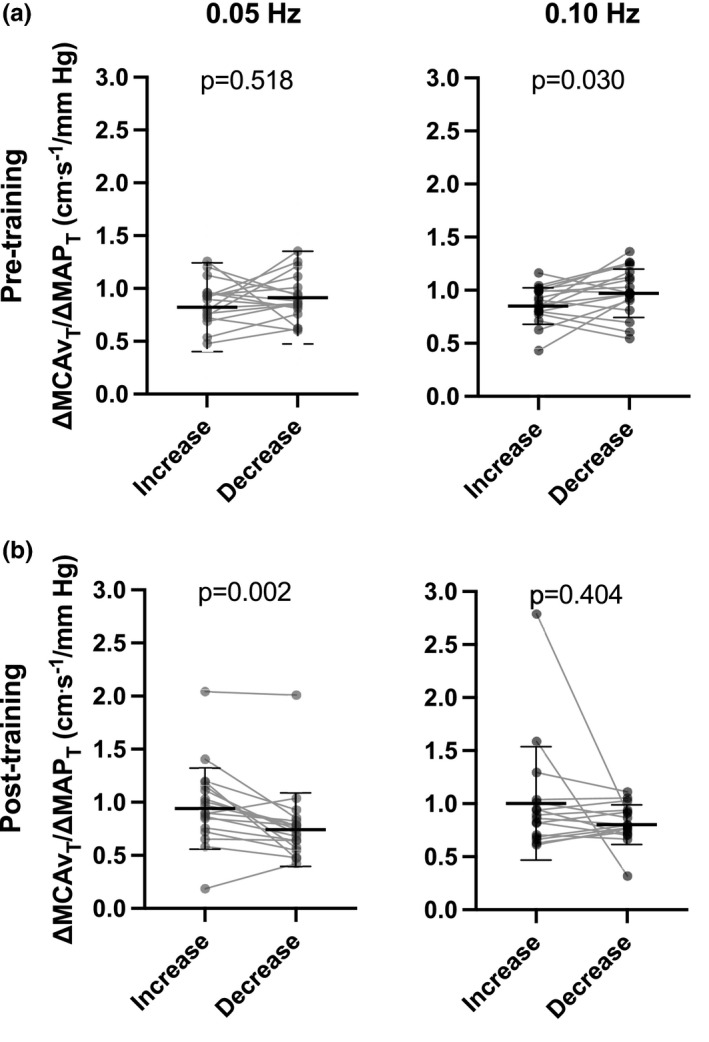
∆MCAv_T_/∆MAP_T_ during transient increases and decrease in mean arterial pressure (MAP). Increases and decreases in MAP during 0.05 Hz (left side) and 0.10 Hz (right side) repeated squat‐stands before (a) and after (b) 6 weeks of high‐intensity interval training to exhaustion. MCA, middle cerebral artery.

ΔMCAv_T_/ΔMAP_T_ was similar during transient increases and decreases in MAP at 0.05 Hz (0.87 ± 0.21 and 0.91 ± 0.22 cm·s^−1^·mmHg^−1^; *p* = 0.518, *d* = 0.16 [CI: −0.73 to 0.35] [negligible]) and smaller during increases in MAP than during decreases in MAP at 0.10 Hz repeated squat‐stands (0.87 ± 0.17 and 0.99 ± 0.23 cm·s^−1^·mmHg^−1^; *p* = 0.030, *d* = 0.56 [CI: 0.15–1.14] [moderate]) (Figure 1).

### 3.4 Influence of HIIT on the directional sensitivity of the cerebral pressure‐flow relationship

Six weeks of HIIT was associated with higher ΔMCAv_T_/ΔMAP_T_ during increases than decreases in MAP at 0.05 Hz repeated squat‐stands (0.97 ± 0.38 and 0.77 ± 0.35 cm·s^−1^·mmHg^−1^; *p* = 0.002, *d* = 0.81 [CI: 0.44–1.39] [large]) and no differences at 0.10 Hz repeated squat‐stands (1.02 ± 0.56 and 0.80 ± 0.18 cm·s^−1^·mmHg^−1^; *p* = 0.404, *d* = 0.36 [CI: −0.13 to 0.63] [small]) (Figure 1). As exploratory analyses, post hoc multiple paired *t*‐test were used to assess the influence of training intensity between the group trained at 85% (*n* = 8) and at 115% (*n* = 10) of maximal aerobic power. ΔMCAv_T_/ΔMAP_T_ was higher during MAP increases in the 85% group (0.91 ± 0.18 and 0.68 ± 0.12, *p* = 0.038, *d* = 1.07 [CI: 0.80–3.62] [large]) but not in the 115% group (1.02 ± 0.50 and 0.85 ± 0.45, *p* = 0.211), although with a moderate effect size (*d* = 0.63 [CI: 0.09–1.50] [moderate]). In contrast, ΔMCAv_T_/ΔMAP_T_ before training was smaller at 0.10 Hz repeated squat‐stands during increases in MAP in the 85% group (0.81 ± 0.19 and 1.04 ± 0.19, *p* = 0.037, *d* = 1.08 [CI: 0.43–3.02] [large]) but similar to decreases in MAP in the 115% group (0.93 ± 0.14 and 0.95 ± 0.26, *p* > 0.999, *d* = 0.16 [CI: −0.98 to 0.57] [negligible]).

## 4 DISCUSSION

To our knowledge, the current study is the first to investigate the impact of HIIT to exhaustion on the directional sensitivity of the cerebral pressure‐flow relationship in endurance‐trained men. The main findings of the current study are two‐fold. First, there was a presence of asymmetry in the cerebral pressure‐flow relationship of our endurance‐trained young men before the training program at 0.10 Hz repeated squat‐stands, where MCAv changes were attenuated when MAP increased. Second, 6 weeks of HIIT to exhaustion led to a disappearance of the asymmetrical response of MCAv with MAP changes at 0.10 Hz, while an inversed pattern appeared at 0.05 Hz repeated squat‐stands. Indeed, the changes in MCAv became less buffered when MAP increased compared with when MAP decreased at this repeated squat‐stand frequency.

### 4.1 Directional sensitivity of the cerebral pressure‐flow relationship before exercise training

During 0.10 Hz, but not 0.05 Hz, repeated squat‐stands, a directional sensitivity of the cerebral pressure‐flow relationship was observed before performing the training program in our participants. We have previously reported such a pattern during 0.10 Hz repeated squat‐stands (i.e., an attenuated change in MCAv when MAP increases compared with when MAP decreases) (Labrecque, Burma, et al., 2022; Labrecque et al., 2021; Roy et al., 2022). This phenomenon might be explained by an increase in cerebral sympathetic nervous activity (SNA) when MAP acutely increases (Cassaglia et al., 2008). Furthermore, Roy et al. (2022) have also demonstrated in their long‐term endurance‐trained group the same pattern of directionality using 0.10 Hz repeated squat‐stands (Roy et al., 2022). Similarly, they showed an absence of a hysteresis‐like pattern at 0.05 Hz repeated squat‐stands. Thus, we are led to believe habitual non‐HIIT specific endurance training is associated with the maintenance of a frequency‐dependent directional sensitivity of the cerebral pressure‐flow relationship in young healthy individuals. On the other hand, we attribute the absence of asymmetrical responses of MCAv at 0.05 Hz repeated squat‐stands to the baroreflex response. The arterial baroreflex is one of the main factors regulating acute fluctuations in MAP (Fisher et al., 2009) and it is frequency‐dependent (Horsman et al., 2013; Zhang et al., 2009). We have previously hypothesized baroreceptors have adequate time to perceive and respond to the slower MAP oscillations during 0.05 Hz repeated squat‐stands (Labrecque et al., 2021).

### 4.2 Impact of HIIT on the directional sensitivity of the cerebral pressure‐flow relationship

The 6‐week HIIT training to exhaustion led to the appearance of an inverted directional sensitivity during 0.05 Hz repeated squat‐stands. Specifically, the change in MCAv became of greater amplitude when MAP increased compared to when MAP decreased at this frequency. This observation is surprising since the opposite has usually been reported in studies observing a directional sensitivity of the cerebral pressure‐flow relationship (i.e. an attenuated change in MCAv when MAP increases compared with when MAP decreases) (Brassard et al., 2017; Labrecque, Burma, et al., 2022; Labrecque et al., 2021; Panerai et al., 2018; Roy et al., 2022; Tzeng et al., 2010).

As discussed in the previous section, the baroreflex response is an important potential factor in the directional sensitivity at 0.05 Hz repeated squat‐stands (Zhang et al., 2009). In terms of cardiorespiratory fitness, midlife (Tomoto et al., 2021) and lifelong (Aengevaeren et al., 2013) aerobic exercise appear to improve baroreflex sensitivity in middle‐aged or elderly athletes, while young endurance‐trained adults tend to show altered baroreflex function (Fadel et al., 2001; Shi et al., 1993). Although not specifically using a HIIT protocol, Uusitalo et al. (2000) reported decreased baroreflex sensitivity after 6–9 weeks of heavy‐volume (7 days a week) endurance training to exhaustion in young female endurance athletes (Uusitalo et al., 2000). Furthermore, Horsman et al. (2014) have shown that baroreflex sensitivity can differ between increases and decreases in MAP (Horsman et al., 2014). Their results indicate similar sensitivity in both directions for 0.05 Hz sit‐to‐stand MAP oscillations in healthy individuals, which may explain the lack of directional sensitivity prior to training at that frequency. However, this may be different if baroreflex sensitivity is indeed decreased after training. Thus, the inverted appearance of directional sensitivity after HIIT during 0.05 Hz repeated squat‐stands could be related to the effects of this type of exercise training on baroreflex sensitivity, specifically in younger adults (Tomoto et al., 2021). Through exploratory analyses, this pattern of directional sensitivity was most remarkable in the lower intensity HIIT group (i.e., 85% of maximal aerobic power). Since both HIIT groups had their training regimen equalized in terms of exhaustion (Paquette et al., 2017) (i.e., more volume to counteract a lesser intensity and vice versa), it could be that the longer training durations were responsible for our finding. Thus far, the physiological explanation for this result remains elusive. Future research is needed to expand these findings to other age‐ranges, cardiorespiratory fitness levels, and clinical conditions.

For 0.10 Hz repeated squat‐stands, 6 weeks of HIIT training negated the presence of directional sensitivity found prior to training. Since SNA appears to play an increasing role in the control of dCA at faster frequencies (i.e., more so at 0.08–0.10 Hz than 0.03–0.05 Hz) (Hamner et al., 2010), we have previously hypothesized SNA to be accountable for the presence of directional sensitivity of the pressure‐flow relationship (Labrecque et al., 2021). Essentially, increased cerebral SNA during increases in MAP (Cassaglia et al., 2008) may be what causes the lower value observed in our metric before HIIT during transient MAP increases induced by 0.10 Hz repeated squat‐stands. Looking at the autonomic effects of exercise training, improved parasympathetic tone and depressed sympathetic modulation of heart rate can be the results of endurance training (Carter et al., 2003), including HIIT (Abreu et al., 2019). Of note, the results from a systematic review on the effects of HIIT on cardiac autonomic control by Abreu et al. (2019) included studies on unfit, sedentary individuals or on clinical populations with cardiovascular diseases and metabolic syndrome (Abreu et al., 2019). Thus, their conclusions may not apply to our endurance‐trained athletes. In female athletes, Coates et al. (2018) indicated an increase in muscle SNA after 3 weeks of overload training, including 1 week of HIIT (Coates et al., 2018). Moreover, short‐term HIIT training in adolescents elicited greater sympathetic predominance after 3 months due to the initial effects of physiological overload and fatigue (McNarry et al., 2019). We could speculate towards similar increases in SNA in our endurance‐trained athletes following 6 weeks of HIIT to exhaustion. However, although there was no autonomic assessment done in the current study, our athletes showed a significant decrease in their resting heart rate (from 54 to 51 bpm), suggestive of further withdrawal of sympathetic tone (Carter et al., 2003). Therefore, a shift of autonomic balance towards parasympathetic tone may help explain our results showing an abolished directionality of dCA at 0.10 Hz repeated squat‐stands post‐training. Another consideration is that cerebral SNA seems to act opposingly to the peripheral circulation, with intracranial pressure influencing sympathetic tone (Guild et al., 2018; Koep et al., 2022). Thus, peripheral measurements of SNA may not be representative of the active sympathetic control of cerebral arteries. On the other hand, since previous studies in humans have suggested SNA‐induced cerebral vasoconstriction during transient decreases in MAP (Ogoh et al., 2008), we cannot exclude a comparable influence of SNA on the regulation of CBF when MAP rapidly increases and decreases. As such, further studies are needed to better understand the effect of SNA on the directional sensitivity of the cerebral pressure‐flow relationship and the autonomic impacts of HIIT in highly fit individuals.

### 4.3 Influence of P_ET_CO_2_ and cerebrovascular reactivity to CO_2_ on the directional sensitivity of the cerebral pressure‐flow relationship

In the current study, over 5 min of repeated squat‐stands, P_ET_CO_2_ increased by +2.7 mmHg pre‐training, and remained constant (−0.2 mmHg) post‐training at 0.05 Hz repeated squat‐stands. For 0.10 Hz repeated squat‐stands, P_ET_CO_2_ increased by +2.7 mmHg pre‐training and by +2.2 mmHg post‐training. We cannot overlook the fact that P_ET_CO_2_ did not remain consistent during repeated squat‐stands. Our rationale is that if these P_ET_CO_2_ changes are similar between each repeated squat‐stands protocol, then at least directional sensitivity metrics become comparable between 0.05 Hz and 0.10 Hz repeated squat‐stands. In regards to the impact of HIIT on P_ET_CO_2_ and cerebrovascular reactivity to CO_2_, the literature is scarce. According to a systematic review by Whitaker et al. (Whitaker et al., 2020), only one study has reported the chronic effect of HIIT on cerebrovascular reactivity to CO_2_. In that study, 12 weeks of HIIT did not influence cerebrovascular reactivity to CO_2_ in breast cancer survivors. We believe the small variations in P_ET_CO_2_ do not explain the changes in dCA behavior in our results. Indeed, Panerai et al. (Panerai et al., 2018) have shown P_ET_CO_2_ had no significant effect in explaining the dCA efficiency between MAP increases and MAP decreases. However, further research is needed to clarify that issue. For example, studies using repeated squat‐stands to examine the directional sensitivity of the cerebral pressure‐flow relationship could be performed with P_ET_CO_2_ clamped at baseline levels.

### 4.4 Effectiveness of dCA

Combining the results from this study with those of our previous work (Drapeau et al.,  2019), we are able to discuss how directional sensitivity may relate to the effectiveness of dCA assessed using TFA‐derived parameters. Indeed, both studies have used the same endurance‐trained participants with the same repeated squat‐stands protocol to evaluate the cerebrovascular impacts of 6 weeks of HIIT to exhaustion, allowing for direct comparison of the two analyses. Drapeau et al. have reported HIIT to exhaustion induces a reduction in TFA phase during 0.10 Hz repeated squat‐stands after 6 weeks, indicative of attenuated dCA (Drapeau et al., 2019). With the current study, this attenuation of dCA appears associated with the currently reported disappearance of directionality in the cerebral pressure‐flow relationship at 0.10 Hz repeated squat‐stands post‐HIIT. This frequency‐dependent association may indicate the presence of directional sensitivity observed before HIIT—specifically when MCAv is better controlled during transient MAP increases—which underlines a state of preserved dCA effectiveness. Although speculative, the presence of the described hysteresis‐like pattern might be beneficial in maintaining adequate cerebral perfusion. In the context of HIIT, directional sensitivity may protect the brain microcirculation from overperfusion during transient MAP surges (Labrecque, Smirl, et al., 2022). On the other hand, Drapeau et al. did not report any impact of HIIT on dCA during 0.05 Hz repeated squat‐stands (Drapeau et al., 2019). Our significant results at 0.05 Hz repeated squat‐stands may thus indicate directional analysis to be a more sensitive method to detect changes in cerebral hemodynamics. Although the inverted directionality pattern at 0.05 Hz repeated squat‐stands could be related to further attenuation of dCA, it may instead reflect alterations related to the effect of HIIT training on baroreflex sensitivity, and not necessarily dCA effectiveness. Further research is warranted to elucidate these notions.

### 4.5 Methodological considerations

Some limitations related to this study need to be acknowledged and further discussed. This study included only endurance‐trained athletes. Accordingly, the results reported herein cannot be extended to other populations. Since sex (Favre & Serrador, 2019; Labrecque et al., 2019) has been reported to affect dCA, it is possible that sex impacts the directional sensitivity of the cerebral pressure‐flow relationship, and thus present results cannot be extended to women. On the other hand, preliminary results using our current analysis suggest there are no differences on our directional metrics between young males and females (Labrecque, Burma, et al., 2022). However, since these results did not include aerobically fit individuals, we cannot exclude an interaction between exercise training and sex on dCA directionality. We used transcranial Doppler ultrasound to measure cerebral blood velocity, which is an accurate estimate of CBF if the diameter of the artery of interest remains constant (Serrador et al., 2000). However, if the diameter changes, the mentioned instrument outputs may overestimate or underestimate CBF during vasodilation and vasoconstriction, respectively. Artery diameter changes have been associated with changes in P_ET_CO_2_ (Lewis et al., 2015; Verbree et al., 2014). However, P_ET_CO_2_ of the first and last five breaths for each squat‐stands have been averaged in the present study and no change was observed pre‐ versus post‐training period. This confirms the MCAv is likely a representative surrogate measure of CBF. Although technical issues prevented P_ET_CO_2_ from being monitored for all participants, it has been measured in most of the participants, and no change was observed. This is in line with our previous studies with healthy participants showing no change in P_ET_CO_2_, thus a similar pattern can be expected from all included participants. Finally, this study did not include a control group of athletes without HIIT. However, it should be noted in a prior investigation that employed repeated squat‐stand maneuvers to assess dCA across multiple study days, there was minimal differences in the TFA derived outcome metrics in the between‐day measures (Burma et al., 2020). Thus, we are confident similar stable findings would hold true in a control group for the current investigation. Therefore, the changes noted in the current findings are most likely attributable to the effects of the HIIT protocol. However, it is also noted the addition of a true control group would have further aided in statistically accounting for the impact of HIIT on the directional sensitivity of the cerebral pressure‐flow relationship, and this should be done in future studies.

## 5 CONCLUSION

The results of the current study indicate the potential of 6 weeks of HIIT to influence the directional sensitivity of the cerebral pressure‐flow relationship in young endurance‐trained men. Specifically, there was a frequency‐dependent effect of HIIT, with the disappearance of directional sensitivity at 0.10 Hz MAP oscillations and the appearance of an inverted directionality pattern at 0.05 Hz MAP oscillations. Further studies investigating the frequency‐dependent mechanisms controlling CBF in response to changes in MAP in endurance‐trained athletes are needed.

## AUTHOR CONTRIBUTIONS

Patrice Brassard contributed to the original idea of the study; Patrice Brassard contributed to data collection; Faezeh Abbariki and Marc‐Antoine Roy contributed to data analyses; Faezeh Abbariki, Marc‐Antoine Roy, and Patrice Brassard contributed to data interpretation; Faezeh Abbariki, Marc‐Antoine Roy, and Patrice Brassard drafted the article. All authors provided approval of the final article.

## FUNDING INFORMATION

Funding of the current project came from the Ministère de l'Education, du Loisir et du Sport du Québec and the Fundation of the Institut Universitaire de Cardiologie et Pneumologie de Québec.

## CONFLICT OF INTEREST

No conflicts of interest, financial or otherwise, are declared by the author(s).
